# Sequencing and analysis of the complete mitochondrial genome of the surgeon fish *Acanthurus leucosternon BENNETT 1833 (Perciformes: acanthuridae)* with phylogenetic consideration

**DOI:** 10.1080/23802359.2016.1197068

**Published:** 2016-11-12

**Authors:** Biji Gurupatham Devadhasan, Prakash Vincent Samuel Gnana, Vladimir Benes

**Affiliations:** a Department of Zoology, Nesamony Memorial Christian College, Marthandam, Tamil Nadu, India;; b Centre For Marine Science and Technology, Manonmaniam Sundaranar University, Rajakkamangalam, Tamil Nadu, India;; c EMBL Heidelberg, Genomics Core Facility, Heidelberg, Germany

**Keywords:** *Acanthurus leucosternon*, mitochondrial genome, phylogeny, Acanthuridae, surgeon fish

## Abstract

In this study, the complete mitochondrial genome of *Acanthurus leucosternon* was determined. The genome is circular, double-stranded DNA molecule with 16,434 bp in length (EU 136032). The structure, gene organization, and gene order is similar as in other vertebrates. The overall base composition is A (29.29%), C (28.53%), T (26.37%), G (15.81%), and with 44.33% G + C content. Overlapping observed between contiguous genes is encoded in the opposite strands. The two 12S and 16S rRNA genes consist of 949 and 1690 nucleotides, respectively. Non-coding control region is 754 bp long and origin of replication is located in the WANCY cluster. Conserved domains in the control regions and secondary structures for the OL regions are also predicted. Molecular phylogenetic analysis using Maximum-Likelihood method of *A. leucosternon* based on the protein-coding gene sequence was also studied.

*Acanthurus leucosternon* is a marine ornamental surgeon fish in the Family Acanthuridae inhabiting coral reefs and sea grass beds. They occur in most tropical and subtropical seas of the world. In India Acanthurus is found throughout the Indian Ocean.

In this present study, the complete mitochondrial genome of *A. leucosternon* was amplified, cloned, and sequenced. Live fishes of *A. leucosternon* were collected from the Bay of Bengal at Rameswaram, Tamilnadu, India. Specimens of *A. leucosternon* (Voucher No: AE05) were preserved in the laboratory museum. The complete circular-double-stranded mitochondrial genome contained a total of 16,434 bp (GenBank accession no EU 136032) with 2rRNAs genes, 13 protein-coding genes, 22 tRNA genes with a control region required for mitochondrial protein synthesis. Most of these genes are encoded by the H-strand, while *ND6* and other eight *tRNAs* in the light (L) strand as in other bony fishes (Inoue et al. 2000). The intergenic spacers of variable lengths of 1–37 nucleotides are also found similar to salmonids (Wilhelm et al. 2003). *Acanthurus leucosternon* mitogenome constitute 70% protein-coding genes, 16rRNA, 9% tRNA, and 5% non-coding regions. The A + T content is high (55.67%) with low G (15.81%) similar to other vertebrates.

The two 12S and 16S rRNA genes consist of 949 and 1690 nucleotides, respectively. The 22 tRNA genes are dispersed along the genome range in size from 65–76 nucleotides, are predicted to fold into the expected clover leaf secondary structure. Overlapping is found between *tRNA^Gln^* and *tRNA^Met^* in a single nucleotide.

*Acanthurus leucosternon* mitogenome have all protein-coding genes and their arrangements similar to other vertebrate mtDNA. Restricted overlapping is observed in the opposite strands between contiguous genes as in most other fishes (Broughton et al. 2001; Vittas et al. 2011), in particular *ATP8-ATP6*, *ND4L-ND4*, and *ND5-ND6* share 10, 7, and 4 nucleotides, respectively.

All protein-coding genes start with ATG. *ND1*, *ATPase 8*, *ND4L*, and *ND5* ends with TAA, *ND6* with TAG and the remaining genes with incomplete TA or T residues. This is quite common and typical among *mtDNA* genes to be completed by post transcriptional polyadenyalation (Ojala et al. 1981). The overall base composition for the protein genes is 69.57%, A + T composition is 55.36%.

The O_L_ was identified as 37bp sequence located between *tRNA^Asn^* and *tRNA^Cys^* in the cluster of WANCY region and sharing 14 bp at 3′ and 5′ end, respectively. The control region of *A. leucosternon* is 754 bp long with rich A + T (66.71%). Several conserved domains such as one termination associated sequence (TAS), one central conserved sequence block and three putative conserved sequence blocks were identified in the control region.

The ML tree was constructed based on the mitochondrial encoded H-strand protein genes of 18 Perciformes fishes. To find the best-fit substitution model using Modeltest version 3.6 program was employed (Posada & Crandall 1998). Based on the resulting ML tree (Figure 1), *A. leucosternon* and *A. lineatus* are grouped together forming sister taxon to the genus Paracanthus and Zebrasoma within the order Acanthuroidei.

**Figure 1.**
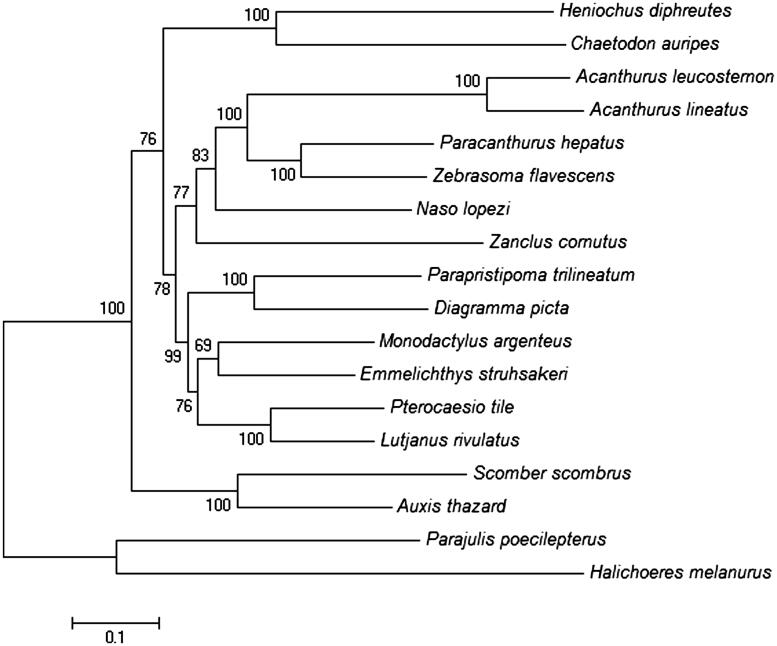
ML tree was generated using the MEGA 6 program based on GTR + I + G model. Initial tree for the heuristic search was obtained by applying the Neighbor-Joining method to a matrix of pairwise distances estimated using the Maximum-Composite Likelihood (MCL) approach. The accession numbers for all 18 species are *Halichoeres melanurus* (AP006018.1), *Chaetodon auripes* (AP006004.1), *A. leucosternon* (EU136032.1), *A. lineatus* (EU273284.2), *Paracanthurus hepatus* (KT826539.1), *Zebrasoma flavescens* (AP006032.1), *Naso lopezi* (AP009163.1), *Zanclus cornutus* (AP009162.1), *Parapristipoma trilineatum* (AP009168.1), *Diagramma picta* (AP009167.1), *Monodactylus argenteus* (AP009169.1), *Emmelichthys struhsakeri* (AP004446.1), *Pterocaesio tile* (AP004447.1), *Lutjanus rivulatus* (AP006000.1), *Scomber scombrus* (AB120717.1), *Auxis thazard* (AB105447.1), *Parajulis poecilepterus* (EF192032.2), and *Heniochus diphreutes* (AP006005.1). There were a total of 10,866 positions in the final dataset. The tree with the highest log likelihood (−102591.12) is shown. The number of nucleotide substitutions per site is indicated by the scale.
